# The Clinical Safety and Efficacy of Sodium Channel Blocker Therapy for Rhythm Control in Atrial Fibrillation: Insights from the REGUEIFA Registry

**DOI:** 10.3390/medsci14010016

**Published:** 2025-12-30

**Authors:** Javier García-Seara, Laila González Melchor, María Vázquez Caamaño, Emilio Fernández-Obanza Windcheid, Raquel Marzoa, Miriam Piñeiro Portela, Eva González Babarro, Pilar Cabanas Grandío, Olga Durán Bobín, Óscar Prada Delgado, Juliana Elices Teja, Evaristo Freire, Mario Gutiérrez Feijoo, Javier Muñiz, Francisco Gude, Carlos Minguito Carazo, Eduardo Barge-Caballero, Carlos González-Juanatey

**Affiliations:** 1 Cardiology Department, University Hospital of Santiago de Compostela, 15706 Santiago de Compostela, Spain; carlos.minguito.carazo@sergas.es; 2 Cellular and Molecular Cardiology Unit, Institute of Biomedical Research of Santiago de Compostela (IDIS-SERGAS), 15706 Santiago de Compostela, Spain; 3 Centro de Investigación Biomédica en Red Enfermedades Cardiovasculares (CIBERCV), Institute of Health Carlos III, 28029 Madrid, Spain; 4 Cardiology Department, University Hospital Lucus Augusti, Institute of Biomedical Research of Santiago (IDIS), 27003 Lugo, Spainolga.duran.bobin@sergas.es (O.D.B.); juliana.elices.teja@sergas.es (J.E.T.); carlos.gonzalez.juanatey@sergas.es (C.G.-J.); 5 Cardiology Department, San Rafel Hospital, 15006 La Coruña, Spain; macrisvc@yahoo.es; 6 Cardiology Department, Hospital Álvaro Cunqueiro and Institute of Health South of Galicia (IISGS), 36312 Vigo, Spain; emilio.fernandez-obanza-windcheid@sergas.es (E.F.-O.W.); pilar.cabanas.grandio@sergas.es (P.C.G.); 7 Cardiology Department, Arquitecto Marcide Hospital, 15405 Ferrol, Spain; raquel.marzoa.fernandez@sergas.es; 8 Cardiology Department, Instituto de Investigación Biomédica de A Coruña (INIBIC), University Hospital of A Coruña, 15006 La Coruña, Spain; miriam.pineiro.portela@sergas.es (M.P.P.); oscar.prada.delgado@sergas.es (Ó.P.D.); eduardo.barge.caballero@sergas.es (E.B.-C.); 9 Cardiology Department, Montecelo University Hospital, 36071 Pontevedra, Spain; eva.gonzalez.babarro@sergas.es; 10 Cardiology Department, Orense University Hospital, 32005 Orense, Spain; evaristo.freire@sergas.es (E.F.); mario.gutierrez.feijoo@sergas.es (M.G.F.); 11 Cardiovascular Research Group, Department of Health Sciences, Instituto de Investigación Biomédica de A Coruña (INIBIC), A Coruña University, Centro de Investigación Biomédica en Red Enfermedades Cardiovasculares (CIBERCV), 15006 A Coruña, Spain; javier.muniz.garcia@udc.es; 12 Research Methodology Group, Institute of Biomedical Research of Santiago (IDIS), University of Santiago de Compostela (USC), 15706 Santiago de Compostela, Spain; francisco.gude.sampedro@sergas.es

**Keywords:** atrial fibrillation, sodium channel blocker, rhythm control, structural heart disease

## Abstract

**Background:** The aim of this study is to assess the safety of sodium channel blocker (SCB) therapy in patients with atrial fibrillation (AF). **Methods:** The REGUEIFA registry is a prospective, observational, multicenter registry from a Community Health Area in Spain that recruited patients with AF, whom it followed for 2 years. **Results:** From the 997 patients, 632 were assigned to a rhythm control strategy and analyzed. Patients exposed to SCBs demonstrated a risk ratio (RR) of 0.38 (95% CI: 0.18–0.79; *p* = 0.007) for worsening heart failure (HF), and 0.40 (95% CI: 0.21–0.78; *p* = 0.005) for the composite endpoint (death, ischemic stroke, or worsening HF), with no significant differences in all-cause mortality, cardiovascular (CV) mortality, ischemic stroke, or bleeding compared with patients not exposed to SCBs. In the subgroup of patients with structural heart disease, no differences were observed between those exposed and those not exposed to SCBs across all the clinical outcomes analyzed (all-cause mortality, CV mortality, ischemic stroke, bleeding and composite event). However, a lower event trend was observed across all these variables. The rate of sinus rhythm at 2 years follow-up was significantly higher in the SCB group (81.8% vs. 63.9%; *p* < 0.001). During Cox regression analysis for all-cause mortality, SCB exposure was not identified as an independent factor (HR: 0.82; 95% CI 0.17–3.87; *p* = 0.802). Age (HR: 1.10; 95% CI: 1.04–1.17; *p* < 0.001) and HF (HR: 4.23; 95% CI: 1.63–11.00; *p* = 0.003) were the only predictors of mortality. **Conclusions:** SCB therapy appears to be safe and effective, both in the overall cohort and in the patient subgroup with AF and structural heart disease. These agents may play a role in AF management in patients with revascularized coronary heart disease, left ventricular hypertrophy, and HF with preserved left ventricular ejection fraction.

## 1. Introduction

An early rhythm control strategy reduces cardiovascular events in patients with atrial fibrillation (AF), with its beneficial effect emerging quickly during follow-up [1,2]. Sodium channel blockers (SCBs) are effective drugs for maintaining sinus rhythm and are associated with a low adverse event rate. A meta-analysis of 60 studies with flecainide showed that 65% of patients were responsive to treatment in the short-term, and 49% in the long-term, indicating its clinical benefit in maintaining sinus rhythm is sustained. Flecainide also reduces the symptoms associated with AF; significantly more patients receiving it reported suppression of palpitations, tachycardia, and chest pain compared with those receiving placebo [3].

Nevertheless, IC antiarrhythmic drugs (AADs) remain underutilized due to the risk of proarrhythmia. CAST I and CAST II trials demonstrated an increase in cardiovascular mortality associated with the use of flecainide, encainide, and moricizine, respectively, in asymptomatic patients with simple ventricular ectopy, myocardial infarction, and left ventricular systolic dysfunction [4,5].

According to the European Society of Cardiology (ESC) guidelines for AF management, flecainide is contraindicated in patients with coronary heart disease (CHD) or reduced left ventricular ejection fraction (LVEF). It should be discontinued if QRS widening exceeds 25% above baseline, or in patients with left bundle branch block or any other conduction block >120 ms. Furthermore, caution is advised in cases with sinoatrial or atrioventricular conduction disturbances. Due to flecainide’s narrow therapeutic margin (0.2–1.0 µg/mL, and proarrhythmia described above >0.7 µg/mL), caution should be exercised in elderly patients and in those with renal impairment. Comparable recommendations are also included in the ACC/AHA/HRS guidelines for AF management [6,7,8].

However, their use in patients with revascularized and stable CHD—a frequent scenario in the contemporary coronary patient management in the era of percutaneous interventions—as well as in patients with heart failure (HF) and preserved LV systolic function, and those with left ventricular hypertrophy (LVH), has not been adequately evaluated.

We analyzed the prognostic impact of SCB use in AF patients at two-year follow-up in the REGUEIFA registry, both in the overall cohort and in those with structural heart disease.

## 2. Material and Methods

### 2.1. Study Design

The REGUEIFA registry is a prospective, observational, multicenter registry from Galicia, a Community Health Area in Northwest Spain. Our study design was as previously described [9]. Briefly, we included patients living in Galicia with AF diagnosis who received a cardiology consultation or were hospitalized in a cardiology ward between January 2018 and February 2020.

The inclusion criteria were age > 18 years, AF > 30 s diagnosed by electrocardiogram or an external or implantable Holter monitor, and an AF episode within the last year before recruitment. AF could be a primary or secondary diagnosis. The included patients signed informed consent forms. The exclusion criteria were reversible AF and participation in an interventional study that examined the frequency of visits and diagnostic tools.

All patients were followed for 2 years, and the collected information included mortality, cardiovascular mortality, cardiovascular hospitalizations, thromboembolic events, bleeding events, quality of life, and changes in rhythm or rate-control strategy. All events were obtained from patients’ electronic clinical history based on outpatient visits with a cardiologist, primary care visits, and emergency attendance. The Galician Society of Cardiology is the registry’s sponsor and promoter, for which the guidelines of the Declaration of Helsinki, the European Union Note for Guidance on Good Clinical Practice CPM/ECH/135/95 and Good Pharmacoepidemiological Practice, and local ethics and norms were followed. The registry was approved by the Ethical Clinical Investigation Committee of Galicia (register number 2016/376) on 15 November 2016.

Patients were defined as having structural heart disease if they presented at least one of the following characteristics:

- History of HF;
- CHD;
- First-degree atrioventricular block;
- Bundle branch block;
- LVH, defined as LV wall thickness ≥ 15 mm.

### 2.2. Statistical Analysis

Continuous variables are described as the mean ± standard deviation (SD) if normally distributed and the median and interquartile range. A *t* test was used forbetween-group comparisons. Categorical variables were expressed as frequencies and percentages, and the χ^2^ test or Fisher’s exact test was applied for between-group comparisons.

The results were expressed with *p*-values, 95% confidence intervals (CIs), and hazard ratios (HRs). Statistical significance was defined by a *p*-value < 0.05. Kaplan–Meier curves were plotted, and survival distributions were compared using the log-rank test.

Single-factor and multivariate Cox proportional hazard analyses were used to identify the prognostic value of SCB drugs in all-cause mortality. For bleeding events, we used a Poisson regression model to take into account recurrence.

A survival analysis with time-dependent exposure was performed. To take into account the fact that treatments change over time—particularly in an observational study like this—and to try to improve the allocation of exposure to each patient, the follow-up time for each treatment was assigned to the corresponding group. Likewise, events were assigned to the treatment group that the patient was receiving at that time. Thus, follow-up was edited according to any change in treatment and restarted in the new group; as a result, some patients contributed to the follow-up of both SCB and non-SCB groups. In no case was the follow-up time duplicated for any patient. The data are treated in this way because if a patient had entered the study at the time of treatment change, they would have been considered in the group with that new treatment. This happened in a total of 99 cases; not doing so would mean assigning these 99 patients to a treatment that they were not receiving at that time. Although this has the advantage of better actual exposure allocation, it is nevertheless carried out under the assumption that the effect of receiving SCB or not does not carry over between periods after the change. The effect on the numbers is that the number of patient observations is 731, 533 patients with 1 record + 99 with 2 records (198), 518 without SCB treatment + 213 with.

## 3. Results

### 3.1. Baseline Characteristics

From the 1001 patients initially recruited in the REGUEIFA registry, 4 were lost during follow-up. We analyzed the 632 patients assigned to a rhythm control strategy. The baseline characteristics are shown in Table 1. Patients treated with SCB drugs were younger; they were also more likely to be men and less frequently presented comorbidities such as hypertension and HF (5.1% vs. 17.3%), although most patients with SCBs and HF had the preserved LVEF form. Patients receiving SCB treatment had less CHD (2.1% vs. 15.6%). Among the 62 patients with CHD not receiving SCB drugs, 43 (69%) had undergone revascularization, whereas among those treated with SCBs, 4 out of 5 patients (80%) had been revascularized. All patients with a history of acute coronary syndrome had been revascularized, with the proportion of non-revascularized cases corresponding to chronic coronary syndrome in both groups.

Regarding AF type, patients with SCBs had fewer first AF episodes and a higher prevalence of persistent AF than patients without SCBs.

Patients exposed to SCBs received baseline AAD twice as often (86.7%) as those not exposed (44%). Among those receiving AAD therapy, flecainide was the most frequently used drug (34.3%), followed by amiodarone (27.8%), and the anticoagulation rate was similar in both groups (Table 1).

### 3.2. Clinical Outcomes

In the overall patient cohort, all-cause mortality was at 3.3%, and the composite endpoint rate of mortality, ischemic stroke, and HF was 13.1%. Among the patients with structural heart disease, all-cause mortality was nearly twice as high (6.1%), and the rate of composite events reached 20.8% at two-year follow-up (Table 2).

In terms of SCB treatment exposure, exposed patients showed significantly lower all-cause mortality (0.85% vs. 4.77%; *p* = 0.01), cardiovascular mortality (0.43% vs. 2.51%; *p* = 0.06), worsening HF (3.85% vs. 14.07%; *p* < 0001), and composite endpoint rates (4.70% vs. 17.59%; *p* < 0001) (Table 3).

When analyzing each follow-up period separately according to SCB exposure, exposed patients demonstrated risk ratios (RRs) of 0.38 (0.18–0.79; *p* = 0.007) in worsening HF and 0.40 (0.21–0.78; *p* = 0.005) in composite event rate without significant differences in all cause-mortality, cardiovascular mortality or ischemic stroke or bleeding (Table 4).

An analysis of cardiovascular events in the subgroup with structural heart disease showed no significant differences between those exposed and those not exposed to SCBs across all the clinical outcomes analyzed (all-cause mortality, cardiovascular mortality, ischemic stroke, bleeding and composite event rate). However, a lower event trend was observed across all these variables: the composite event rate was 22.6% in those not exposed to SCBs vs. 11.9% in those exposed (Table 5).

In the Cox regression analysis for all-cause mortality, SCB exposure was not identified as an independent factor (HR: 0.82; 95% CI 0.17–3.87; *p* = 0.802). Age (HR: 1.10; 95% CI: 1.04–1.17; *p* < 0.001) and HF (HR: 4.23; 95% CI: 1.63–11.00; *p* = 0.003) were the only predictors of mortality, and not CHD (HR: 1.35; 95% CI: 0.48–3.81; *p* = 0.568) and LVH (HR: 0.97; 95% CI: 0.36–2.63; *p* = 0.947) (Table 6).

**Figure 1:**
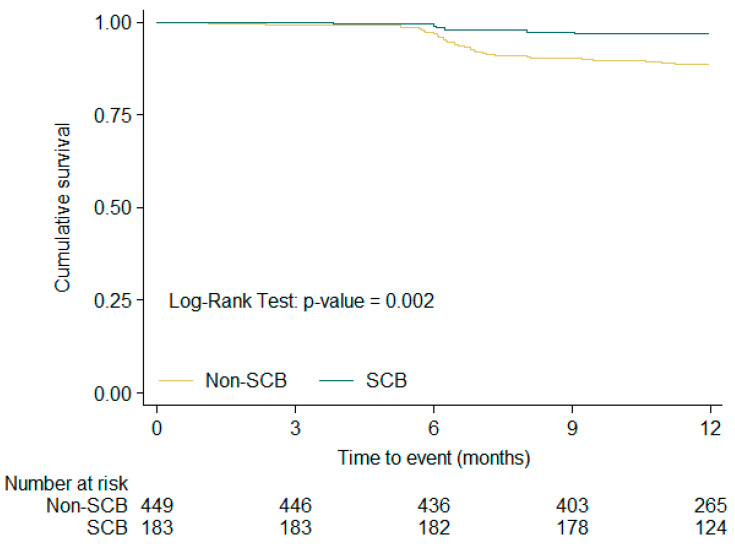
Kaplan–Meier composite event free-survival curves according SCB exposure or non-exposure in the Baseline—V1 period.

**Figure 2:**
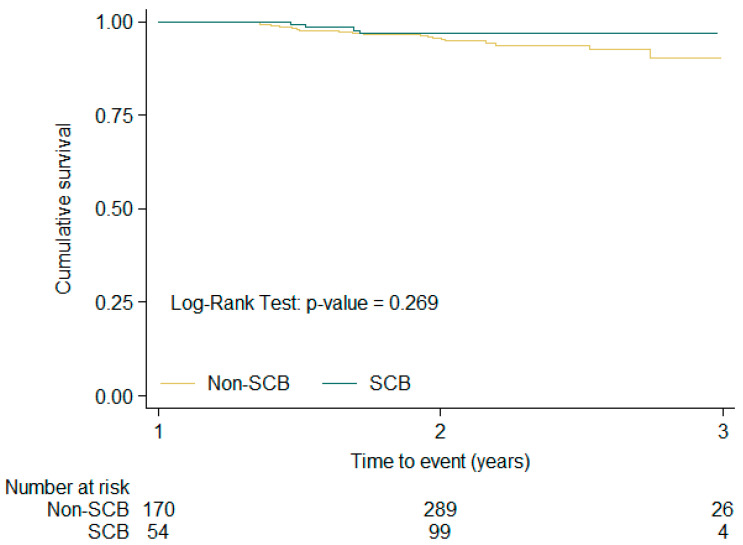
Kaplan–Meier composite event free-survival curves according to SCB exposure or non-exposure in the V1–V2 period.

**Figure 3:**
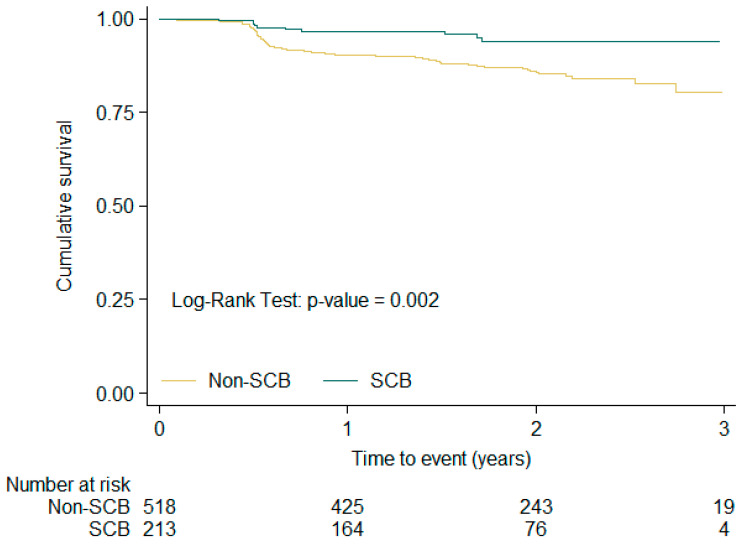
Kaplan–Meier combined event free-survival curves according SCB exposure or non-exposure in the 2-year follow-up period.

Figure 1, Figure 2 and Figure 3 show the Kaplan–Meier free-survival curves for the composite event at 1-year, 1–2-year, and overall follow-up, according to SCB exposure or non-exposure. In the overall analysis, patients exposed to SCBs had a significantly lower composite event rate (log-rank *p* value: 0.002).

**Figure 4:**
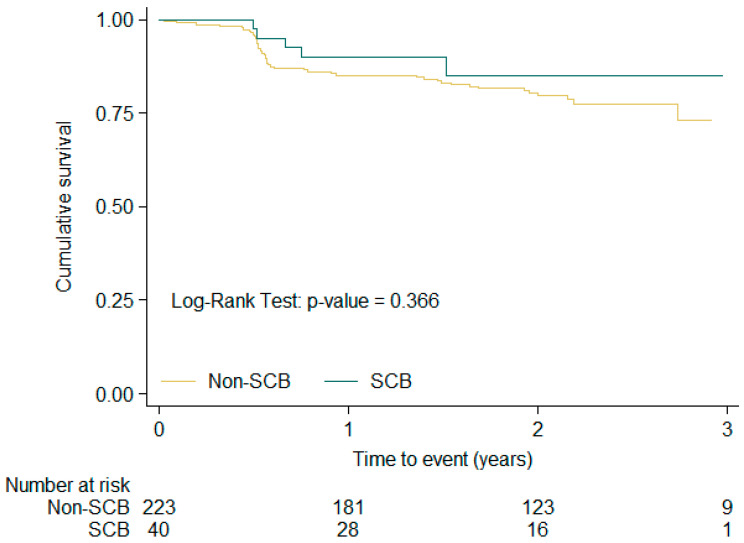
Kaplan–Meier combined event free-survival curves according to SCB exposure or non-exposure in the subgroup with structural heart disease in the 2-year follow-up period.

Figure 4 shows the same curves for the composite event in the subgroup with structural heart disease, comparing those exposed to SCBs with those not exposed. No significant differences were observed between the two groups (log-rank *p* value: 0.366).

## 4. Discussion

The principal finding of this study is that SCBs appear to be safe for managing patients with AF, both in the overall cohort and in the structural heart disease subgroup. These observations are derived from the REGUEIFA registry, which encompasses AF patients managed by cardiologists in both in- and outpatient settings across the healthcare area of Galicia in Northwestern Spain. SCB treatment was associated with a substantially lower incidence of new-onset or worsening HF compared with non-exposure, yielding an estimated 62% relative risk reduction. A consistent effect was observed for the composite outcomes of death, ischemic stroke or hospitalization for HF, corresponding to a 60% relative risk reduction.

### 4.1. SCBs in the Global Population

In the EAST-AFNET 4 trial, SCB-treated patients assigned to rhythm control experienced fewer primary efficacy endpoint events (3.0 per 100 compared with 4.9 per 100 patient-years in those who never received SCBs (*p* < 0.001)). SCB therapy was identified as an independent predictor of survival in the Cox regression analysis (HR: 0.55; 95% CI 0.39–0.77; *p* < 0.001) [10]. In the REGUEIFA registry, patients under rhythm control with SCB therapy showed a composite endpoint incidence rate of 2.87 per 100 patient-years (95% CI 1.55–5.34), whereas in non-exposed patients this was 7.17 per 100 patient-years (95% CI 5.70–9.02), resulting in an RR for exposed versus non-exposed patients of 0.40 (95% CI 0.21–0.78; *p* = 0.005). Therefore, despite a shorter follow-up period (2 vs. 5.1 years in EAST-AFNET 4) and a more heterogeneous AF population (in EAST-AFNET 4, the time since AF diagnosis was required to be <1 year), our results show similar clinical outcomes. These findings further reinforce the benefits of SCBs as preferred agents for maintaining sinus rhythm across a broad spectrum of patients with AF.

### 4.2. SCBs in the Structural Heart Disease Population

However, the use of SCBs is limited by their contraindication in patients with structural heart disease, which introduces an inherent selection bias. The CAST and CAST II trials demonstrated that SCB therapy (flecainide, moricizine, and encainide) was associated with a 2.5-fold increase in mortality in patients with prior myocardial infarction and a high premature ventricular contraction burden [4,5]. In addition, mortality was significantly higher in patients with non-Q-wave infarction compared with those with Q-wave infarction, with a five-fold higher relative risk of death. As such, the relative risk associated with encainide or flecainide compared to a placebo in non-Q-acute myocardial infarction patients was 8.7, significantly higher than the 1.7 observed for Q-acute myocardial infarction patients (*p* = 0.03). This suggests that a drug–ischemia interaction, rather than a drug–scar interaction, mediates the risk of death, supporting the presence of a link between ischemia and electrical instability [11]. In fact, in further support of this hypothesis, it should be noted the European Rythmol/Rytmonorm AF Trial (ERAFT), which included patients with previous myocardial infarction but without unstable angina, did not demonstrate an increase in mortality and ventricular arrhythmias in patients treated with propafenone (structural heart disease at 42% in the propafenone group vs. 36% in placebo) [12,13].

The CAST II trial included an early phase of analysis at 14 days post-myocardial infarction to evaluate the efficacy of suppressing ventricular ectopy with moricizine and assess cardiovascular events following the publication of CAST I. The study was terminated due to the detection of increased mortality during this early phase and the low probability of survival benefit in long-term follow-up. This indicates that, in the setting of non-revascularized myocardial infarction with reduced LVEF and acute ischemia, the proarrhythmic effect occurs early. It also suggests that the benefit of AAD in asymptomatic patients with simple ventricular ectopy is limited [5,11]. Indeed, the CAST trial did not include patients with complete revascularization of the culprit lesion, as this would potentially reduce pro-arrhythmic post-myocardial infarction events and long-term mortality, as well as potentially causing significant ventricular post-myocardial infarction dysfunction over time. Furthermore, the increased mortality described in the CAST trial was mostly related to the flecainide-induced excess of proarrhythmic events in elderly patients with significant pre-existing cardiovascular comorbidity [13,14]. These findings led the U.S. Food and Drug Administration to recommend that flecainide be contraindicated in all patients with structural heart disease, regardless of etiology.

However, our results suggest that the safety concerns derived from the CAST era may not be fully applicable to contemporary AF populations without active ischemia, particularly in patients with stable structural heart disease and preserved or mildly reduced LV function [15]. In our series, all patients presenting with acute coronary syndrome underwent revascularization. All non-revascularized patients belonged to the category of chronic coronary syndrome. Another clinically relevant difference lies in concomitant medication. In the CAST trial, only 27% of patients were treated with β-blockers, whereas this proportion increased to approximately 50% in a series of patients with stable CHD [16], and reached 78.6% in our cohort.

Recent analyses from non-randomized cohorts have demonstrated the safety of IC agents in structural heart disease [16,17,18,19]. Compared with class III antiarrhythmic therapy, flecainide was not associated with an increased risk of proarrhythmia or HF events in patients with stable or revascularized CHD [17]. In a large retrospective study including 3444 patients treated with a class IC AAD for atrial tachycardia and 2216 treated with sotalol or dofetilide, class IC therapy was independently associated with improved overall survival; furthermore, class IC agents proved safe in patients with non-obstructive CHD. However, a significant interaction emerged between IC agent use and the extent of CHD, with patients presenting obstructive lesions showing poorer event-free survival (HR 3.80; 95% CI 1.67–8.67; *p* = 0.002) compared with class III agents (sotalol). No significant interaction was observed between class IC agent use and HF presence in this cohort [18]. In another series of patients receiving SCB therapy, CHD was present in up to 60% of cases, with obstructive lesions identified in 17.3% (91.2% of which involved two or more vessels). Remarkably, no ventricular arrhythmias were observed during long-term follow-up (mean 4.1 years). Flecainide use was not associated with an increase in either all-cause mortality or ventricular arrhythmias among patients with perfusion defects detected by SPECT, or those with CHD determined by invasive angiography and CT coronary angiography. In patients taking flecainide, multivessel CHD involvement, degree of stenosis, or flecainide dose were not associated with increased mortality [16]. Finally, in a limited population of AF patients with preserved left ventricle function and positron emission tomography–coronary flow capacity, indicating occult CHD, IC AAD treatment does not appear to increase mortality [19].

Another issue that warrants further analysis is the use of SCBs in patients with structural heart diseases other than CHD [10,20,21]. The efficacy and safety of SCBs were evaluated in a subanalysis of the EAST-AFNET 4 trial, which examined a subgroup of patients treated with SCBs with structural heart disease defined as stable HF (26%), CHD (6%, including prior myocardial infarction, coronary artery bypass grafting, or percutaneous coronary intervention), LVH (4%) and abnormal LVEF (6%). The safety endpoint (death, hospitalization for HF, and rhythm control-related adverse events) was lower—although not statistically significant—in the SCB group (15.2%) compared with the non-SCB group (19.9%). The incidence of torsade de pointes was extremely low, with only one patient (0.4%) in the SCB group. In terms of atrioventricular block (AVB), drug-induced toxicity, or pharmacologically induced bradycardia, no significant differences were observed between patients treated with SCB and those not [10,20]. Another series evaluated the safety of flecainide in patients with structural heart disease (28% LV hypertrophy, 25% impaired LVEF, and 10.6% with CHD). The incidence of ventricular tachycardia or ventricular fibrillation was not higher among patients with structural heart disease receiving flecainide therapy [21]. Both findings are consistent with the results obtained in our series, where the composite endpoint incidence was higher in the non-SCB group (22.6%) compared with the SCB-treated group (11.9%); however, this difference did not reach statistical significance.

Finally, flecainide has also proven useful in managing arrhythmias other than AF in patients with non-ischemic structural heart disease. As such, Hyman et al. demonstrated the safety and efficacy of class IC antiarrhythmic drugs in suppressing premature ventricular contractions (PVCs); these drugs led to LVEF recovery without the occurrence of sustained ventricular arrhythmias in patients with PVC-induced cardiomyopathy for whom catheter ablation had been unsuccessful [22]. Similarly, Ermakov et al. demonstrated that the addition of flecainide with sotalol or metoprolol was effective in managing recurrent ventricular arrhythmias in patients with arrhythmogenic right ventricular cardiomyopathy [23].

Additionally, it should be stressed that the median age of patients treated with class IC agents in the EAST-AFNET 4 trial was 69 years, with only 25% of the patients being >75 years; as such, this limits the generalizability of the study’s findings to the large amount of very old patients presenting AF in the real world, who are often affected by frailty and many comorbidities [15,24,25]. In our series, the mean age of patients receiving SCB therapy was even lower (58 vs. 65 years in those not treated with SCBs), indicating that these results should be interpreted with particular caution in older patients. Another difference that may have clinical relevance is that, in the EAST-AFNET 4 study, the rate of sinus rhythm at 2 years was similar between patients exposed to SCBs and those not (88% vs. 82%). In contrast, in our series, this rate was significantly higher among patients treated with SCBs (82% vs. 64%; *p* < 0.001), which may at least partially account for the clinical benefits associated with SCB therapy.

In the real world, the use of class IC AADs in settings such as hypertension with LVH, or in selected patients with stable HFpEF or stable CHD—also called chronic coronary syndromes—is not uncommon, as highlighted by observational studies [16,17,18] and surveys [20,26]. This suggests that non-adherence to guideline recommendations occurs quite frequently both in Europe and the USA. Although current guidelines discourage their use in this setting, a recent study from the ORBIT-AF registry showed that adherence to guideline recommendations for AF management was lowest (56%) among patients receiving class IC AAD, despite having known CHD [27]. Class IC AAD represents a cornerstone in AF therapy and all the data presented here should prompt us to consider that some deviation may be reasonable in selected patients with thorough risk–benefit ratio assessment and careful patient monitoring [15,28].

### 4.3. Limitations

The results of the REGUEIFA study may potentially be influenced by a certain center-dependent effect, as the volume of included patients varied among different recruiting centers. Although the consecutiveness of the included patients was pursued among recruiters, it could not be fully guaranteed. Survival analysis with time-dependent exposure was conducted as follows: for each patient, the follow-up period was split into two observation intervals, one spanning from the baseline visit to the 1-year follow-up assessment and a second from the 1- to 2-year follow-up visit.

## 5. Conclusions

SCB therapy appears to be safe and effective, both in the overall cohort and in subgroups of patients with AF and structural heart disease. These agents may play a role in managing AF in patients with revascularized coronary artery disease, LVH, and HF with preserved left ventricular ejection fraction.

## Figures and Tables

**Table 1:** Comparison of baseline characteristics between patients with vs. without SCB treatment during follow-up.

Variable	Not Exposed to SCB n = 398	%/Mean ± SD	Exposed to SCB n = 234	%/Mean ± SD	*p*-Value
**Basic patient information**					
Age (years)	398	65.5 ± 10.5	234	58.6 ± 10.1	<0.001
Women	127	31.9	55	23.5	0.024
BMI (kg/m^2^)	395	29.9 ± 5.0	234	29.7 ± 4.7	0.738
**Patient history**					
Renal disease	17	4.3	6	2.6	0.268
Hypertension	239	60.0	119	50.8	0.024
Heart failure	69	17.3	12	5.1	<0.001
*Preserved LVEF > 50%*	22	31.9	7	58.3	
*Reduced LVEF < 40%*	31	44.9	2	16.7	
*Mid-range LVEF 40–50%*	14	20.3	2	16.7	
Coronary artery disease	62	15.6	5	2.1	<0.001
Previous AMI	43	7.1	3	1.2	<0.001
*NSTEMI*	29	6.5	2	1.0	
*STEMI*	14	3.1	1	0.5	
Stable angina	19	4.7	2	1.0	
Previous coronary revascularization					
Surgical	6	1.5	2	0.8	
Percutaneous	37	9.3	2	0.8	
Any previous coronary revascularization	43	10.8	4	1.7	<0.001
**AF characteristics**					
Clinical AF type					<0.001
First diagnosis	121	30.4	27	11.5	
Paroxysmal	115	28.9	87	37.2	
Persistent	131	32.9	113	48.3	
Long-standing persistent	14	3.5	7	3.0	
Permanent	17	4.3	0	0.0	
Time since first AF episode (months), mean ± SD	277	40.8 ± 64.5	206	39.0 ± 58.0	0.396
If not permanent, mean episode duration (hours), mean ± SD	259	1543.2 ± 3356.7	205	1387.5 ± 4407.1	0.351
**Baseline ECG and follow-up**					
Sinus rhythm	129	32.4	108	46.1	0.001
Two-year sinus rhythm	234	63.9	184	81.8	<0.001
First-degree AV block	13	3.3	6	2.6	0.618
Bundle branch block	31	7.8	1	0.4	<0.001
RBBB	16/31	51.6	1/1	100.0	1.000
LBBB	14/31	45.2	0/1	0.0	1.000
**Previous strategy**					
First AF diagnosis	179	45.0	71	30.3	<0.001
Rhythm control	192/219	87.7	157/163	96.3	0.003
Rate control	55/219	25.1	13/163	8.0	<0.001
Pharmacological cardioversion	50/192	26.0	50/157	31.8	0.233
Electrical cardioversion	104/192	54.2	87/157	55.4	0.816
Any previous cardioversion	125/192	65.1	111/157	70.7	0.266
**AF ablation**					0.586
Pulmonary vein isolation	41	21.3	32	20.4	
AV node ablation	2	1.0	0	0.0	
**Additional tests**					
LVEF (%), mean ± SD	273	54.4 ± 13.2	120	61.1 ± 7.3	<0.001
LVH	114/376	30.3	22/221	9.9	<0.001
**Medication**					
Beta-blockers	275	69.1	184	78.6	0.009
Digoxin	15	3.8	0	0.00	0.002
ACEI/ARB	209	52.5	92	39.3	0.001
Aldosterone antagonists	48	12.1	3	1.3	<0.001
Diuretics	131	32.9	23	9.8	<0.001
Statins	190	47.7	81	34.6	0.001
Oral antidiabetics	58	14.6	18	7.7	0.010
Antiplatelet therapy	20	5.0	2	0.8	0.006
Anticoagulation	355	89.2	206	88.0	0.655
Baseline AAD	175	44.0	203	86.7	<0.001
*AAD follow-up*					
Amiodarone	176	27.8			
Dronedarone	5	0.7			
Sotalol	13	2.0			
Ranolazine			1	0.1	
Flecainide			217	34.3	
Propafenone			22	3.4	

AMI: acute myocardial infarction; NSTEMI: non-ST elevation myocardial infarction; STEMI: ST elevation myocardial infarction; AF: atrial fibrillation; AV: atrioventricular; RBBB: right bundle branch block; LBBB: left bundle branch block; LVEF: left ventricular ejection fraction; LVH: left ventricular hypertrophy; ACEI/ARB: angiotensin-converting enzyme inhibitors/angiotensin receptor blockers; AAD: antiarrhythmic drugs.

**Table 2:** Events throughout follow-up. A. Global cohort. B. Structural heart disease cohort.

Event	n	%
A. 632		
All-cause mortality	21	3.32
Cardiovascular mortality	11	1.74
Ischemic stroke	6	0.95
Worsening heart failure	65	10.28
Composite event	83	13.13
B. 245		
All-cause mortality	15	6.12
Cardiovascular mortality	8	3.26
Ischemic stroke	3	1.22
Worsening heart failure	39	15.92
Composite event	51	20.82

Bleeding is a recurrent event. There were 35 bleeding episodes in 29 different patients. Composite event (mortality, ischemic stroke, or worsening heart failure, whichever occurred first).

**Table 3:** Comparison of events between patients with vs. without SCB treatment during follow-up.

	No SCB (n = 398)		Yes SCB (n = 234)		*p*-Value
	n	%	n	%	
All-cause mortality	19	4.77	2	0.85	0.010
Cardiovascular mortality	10	2.51	1	0.43	0.062
Worsening of HF	56	14.07	9	3.85	<0.001
Composite event	70	17.59	11	4.70	<0.001

SCB: sodium channel block. Composite event (mortality, ischemic stroke, or worsening heart failure).

**Table 4:** Incidence rate (per 100 person-years) of events of interest according to SCB exposure.

Event	Period	All Patients—Patient-Years	All Patients—No. Events	All Patients—IR (95% CI)	Not Exposed to SCB—Patient-Years	Not Exposed to SCB—No. Events	Not Exposed to SCB—IR (95% CI)	Exposed to SCB—Person-Years	Exposed to SCB—No. Events	Exposed to SCB IR (95% CI)	RR (95% CI)	*p*-Value
All-cause mortality	Baseline—V1	672.2	9	1.34 (0.70–2.57)	475.8	8	1.68 (0.84–3.36)	196.4	1	0.51 (0.07–3.61)	0.30 (0.04–2.42)	0.232
	V1–V2	693.9	12	1.73 (0.98–3.05)	542.5	11	2.03 (1.12–3.66)	151.4	1	0.66 (0.09–4.69)	0.33 (0.04–2.52)	0.258
	Total	1366.0	21	1.54 (1.00–2.36)	1018.2	19	1.87 (1.19–2.93)	347.8	2	0.57 (0.14–2.30)	0.31 (0.07–1.35)	0.100
Cardiovascular mortality	Baseline—V1	672.2	7	1.04 (0.50–2.18)	475.8	6	1.26 (0.57–2.81)	196.4	1	0.51 (0.07–3.61)	0.40 (0.05–3.35)	0.385
	V1–V2	693.9	4	0.58 (0.22–1.54)	542.5	4	0.74 (0.28–1.96)	151.4	0	0 (–)	0 (–)	0.291
	Total	1366.0	11	0.81 (0.45–1.45)	1018.2	10	0.98 (0.53–1.83)	347.8	1	0.29 (0.04–2.04)	0.27 (0.03–2.20)	0.189
Ischemic stroke	Baseline—V1	672.2	4	0.60 (0.22–1.59)	475.8	4	0.84 (0.32–2.24)	196.4	0	0 (–)	0 (–)	0.199
	V1–V2	693.9	2	0.29 (0.07–1.15)	542.5	2	0.37 (0.09–1.47)	151.4	0	0 (–)	0 (–)	0.455
	Total	1366.0	6	0.44 (0.20–0.98)	1018.2	6	0.59 (0.26–1.31)	347.8	0	0 (–)	0 (–)	0.138
Worsening of HF	Baseline—V1	672.2	47	6.99 (5.25–9.31)	475.8	42	8.83 (6.52–11.95)	196.4	5	2.55 (1.06–6.12)	0.29 (0.11–0.73)	0.005
	V1–V2	693.9	18	2.59 (1.63–4.12)	542.5	15	2.77 (1.67–4.59)	151.4	3	1.98 (0.64–6.14)	0.72 (0.21–2.47)	0.596
	Total	1366.0	65	4.76 (3.73–6.07)	1018.2	57	5.60 (4.32–7.26)	347.8	8	2.30 (1.15–4.60)	0.38 (0.18–0.79)	0.007
Bleeding	Baseline—V1	672.2	16	2.38 (1.46–3.89)	475.8	15	3.15 (1.90–5.23)	196.4	1	0.51 (0.07–3.61)	0.16 (0.02–1.22)	0.043
	V1–V2	693.9	19	2.74 (1.75–4.29)	542.5	15	2.77 (1.67–4.59)	151.4	4	2.64 (0.99–7.04)	0.96 (0.32–2.88)	0.935
	Total	1366.0	35	2.56 (1.84–3.57)	1018.2	30	2.95 (2.06–4.21)	347.8	5	1.44 (0.60–3.45)	0.50 (0.20–1.26)	0.135
Composite event	Baseline—V1	672.2	56	8.33 (6.41–10.83)	475.8	50	10.51 (7.97–13.87)	196.4	6	3.05 (1.37–6.80)	0.29 (0.12–0.68)	0.002
	V1–V2	693.9	27	3.89 (2.67–5.67)	542.5	23	4.24 (2.82–6.38)	151.4	4	2.64 (0.99–7.04)	0.62 (0.22–1.80)	0.378
	Total	1366.0	83	6.08 (4.90–7.53)	1018.2	73	7.17 (5.70–9.02)	347.8	10	2.87 (1.55–5.34)	0.40 (0.21–0.78)	0.005

HF: heart failure; IR: incident rate; RR: risk ratio between those exposed to SCBs and those not. Composite event (mortality, ischemic stroke, or worsening heart failure).

**Table 5:** Events in those with structural heart disease comparing patients with and without SCB treatment during follow-up.

n = 245	Not Exposed to SCB (n = 203)		Exposed to SCB (n = 42)		*p*-Value	
Event	n	%	n	%		
All-cause mortality	14	6.90	1	2.38	0.479	
Cardiovascular mortality	8	3.94	0	0.00	0.358	
Worsening HF	35	17.24	4	9.52	0.254	
Composite event	46	22.66	5	11.90	0.118	

HF: heart failure. Composite event (mortality, ischemic stroke, or worsening heart failure).

**Table 6:** Cox regression analysis for all-cause mortality.

Variable	HR	95% CI		*p*-Value
		Lower	Upper	
Unadjusted model (n = 632 patients; 21 events)				
Exposed to SCB	0.32	0.07	1.36	0.122
Adjusted model (n = 597 patients; 19 events)				
Exposed to SCB	0.82	0.17	3.87	0.802
Age	1.10	1.04	1.17	<0.001
Sex	0.42	0.13	1.29	0.129
Heart failure	4.23	1.63	11.00	0.003
Coronary heart disease	1.35	0.48	3.81	0.568
Left ventricular hypertrophy	0.97	0.36	2.63	0.947

SCB: sodium channel blocker. HR: hazard ratio.

## Data Availability

The original contributions presented in this study are included in the article. Further inquiries can be directed to the corresponding author.
